# Effects of initial graft tension on clinical outcome after anatomic double-bundle anterior cruciate ligament reconstruction: comparison of two graft tension protocols

**DOI:** 10.1186/s12891-016-0909-y

**Published:** 2016-02-09

**Authors:** Eiji Kondo, Kazunori Yasuda, Nobuto Kitamura, Jun Onodera, Masashi Yokota, Tomonori Yagi, Norimasa Iwasaki

**Affiliations:** The Department of Advanced Therapeutic Research for Sports Medicine, Hokkaido University Graduate School of Medicine, Kita-15 Nishi-7, Kita-ku, Sapporo, 060-8638 Japan; The Department of Sports Medicine and Joint Surgery, Hokkaido University Graduate School of Medicine, Sapporo, Hokkaido Japan; The Department of Orthopaedic Surgery, Yagi Orthopaedic Hospital, Sapporo, Hokkaido Japan; The Department of Orthopaedic Surgery, Hokkaido University Graduate School of Medicine, Sapporo, Hokkaido Japan

**Keywords:** Anterior cruciate ligament, Anatomic reconstruction, Double bundle, Graft fixation, Hamstring tendon, Tension

## Abstract

**Background:**

In anatomic double-bundle anterior cruciate ligament (ACL) reconstruction, there are great controversies concerning the ideal graft tension protocols. The purpose of this study was to clarify differences in the effect of two graft tension protocols on the clinical outcome after anatomic double-bundle anterior cruciate ligament (ACL) reconstruction by comparing the minimum 2-year clinical results.

**Methods:**

Ninety-seven patients with unilateral anatomic double-bundle ACL reconstruction were divided into two groups. In the first 44 patients (Group I), a 40-N tension was applied to each of the two hamstring autografts at 30° of knee flexion, and simultaneously fixed onto the tibia. In the remaining 53 patients (Group II), a 30-N tension was applied to each graft at 10° of knee flexion, and simultaneously fixed onto the tibia. Each patient was examined 2 years after surgery.

**Results:**

There wasn’t a significant difference in the background of the two groups. There was no significant difference in the postoperative anterior laxity between the two groups. The average was 1.1 mm and 0.9 mm in Groups I and II, respectively. There wasn't any differences between the two groups in Lysholm knee score, International Knee Documentation Committee (IKDC) evaluation and muscle strength. Four patients had loss of knee extension in a range of 5° and 10° in Group I and none of the patients in Group II exhibited any loss in knee extension; which was statistically significant (*p* = 0.025).

**Conclusion:**

The two initial graft tension protocols did not result in any significant differences in the Lysholm knee score and IKDC grade. However, it was noted that the 40-N tension applied to each graft at 30° of knee flexion more significantly induced loss of knee extension in comparison to the 30-N tension applied to each graft at 10°. From a clinical viewpoint, the loss of knee extension is one of the pathological conditions that should be absolutely avoided after ACL reconstruction. Therefore, the 30-N tension applied to each graft at 10° is preferable to the other graft tension protocol.

## Background

Anterior cruciate ligament (ACL) reconstruction is a routine procedure in the field of knee surgery. Although the single-bundle ACL reconstruction procedure remains the gold standard, anatomic double-bundle ACL reconstruction procedures have recently attracted a great deal of attention due to their in vitro biomechanical advantages [1–5]. In both ACL reconstruction procedures, the graft tension technique, which includes applying a certain magnitude of initial tension to a graft and fixing the graft at a certain degree of knee flexion, has been recognized as one of the most important variables [5–11], because it will have impacts on the outcome of the surgery. For single-bundle ACL reconstruction procedures, several investigators recommend relatively high and low initial tension in a range between 20 and 90 N to obtain better knee stability at 2 years postoperatively [12–15]. Cunningham et al described how most surgeons apply “sufficient magnitude” of initial tension, which is in a range of 40 to 90 N, to a graft at full extension or a slightly flexed position [16].

In anatomic double-bundle ACL reconstruction, however, there are great controversies concerning the ideal graft tension protocol [17–19]. Many investigators have tried to apply various combinations of initial tension magnitudes to the anteromedial (AM) and posterolateral (PL) bundle grafts at different angles of knee flexion [19–26]. In these studies, the clinical outcome, specifically the postoperative knee stability, differed significantly. Therefore, because there is a possibility that initial graft tension protocol significantly affects the clinical outcome after anatomic double-bundle ACL reconstruction, there is an urgent need to clarify the application of initial graft tension in the field of ACL reconstruction. However, there had been no clinical outcome studies in which two initial graft tension protocols were compared in patients who underwent the same anatomic double-bundle ACL reconstruction procedure.

Thus, the authors conducted a prospective comparative cohort study using a total of 107 patients who underwent identical anatomic double-bundle ACL reconstruction performed by the same surgeon. In this study, the authors used two different tension protocols based on the author’s in vivo biomechanical study [5]: in one a 40-N initial tension was applied to each graft at 30° of knee flexion, and in the other a 30-N initial tension was applied to each graft at 10° of knee flexion. In each technique, the 2 grafts were simultaneous firmly fixed onto the tibia thereafter. The purpose of this study was to clarify differences in the effect of these two graft tension protocols on the clinical outcome after anatomic double-bundle ACL reconstruction by comparing the minimum 2-year clinical results.

## Methods

### Study design

A prospective comparative cohort study was carried out using a total of 107 patients, who underwent anatomic double-bundle ACL reconstruction in our hospital between 2008 and 2011 using the same surgical procedure except for the graft tension protocol. The study was approved by the institutional review board in our hospital prior to commencement (Institutional Review Board in Hokkaido University Hospital (009-0164)). All patients were informed that if they did not want to be in this study, they could choose another ACL reconstruction procedure. All investigations were conducted in conformity with ethical principles of research, and that informed consent was obtained. Between 2008 and 2009, 49 patients underwent anatomic double-bundle ACL reconstruction, in which a 40-N tension was applied to both the AM and PL bundle grafts at 30° of knee flexion and simultaneously fixed onto the tibia (Group I). Between 2010 and 2011, the remaining 58 consecutive patients underwent anatomic double-bundle ACL reconstruction, in which a 30-N tension was applied to each graft at 10° of knee flexion and simultaneously fixed onto the tibia (Group II). All surgeries were performed by one senior orthopaedic surgeon (K.Y.) using the same procedures reported previously [24]. Each patient showed chronic ACL-deficiency in the unilateral knee at the time of surgery. The diagnosis of injured ligaments was made based on a detailed history of the knee injury, physical examination on pathologic status and abnormal laxity, routinely performed plain radiographs and magnetic resonance imaging scans, and the findings at surgery. Patients with a combined ligament injury, a concurrent fracture, a history of any previous operations on the knee, or knee osteoarthritis were excluded from this study. The time from onset of injury to surgery was 1 month or more. At the time of reconstruction, the medial or lateral meniscus was partially resected in 22 patients (12 in Group I, and 10 in Group II), and repaired in seven patients (2 in Group I, and 5 in Group II) (Table 1). No patients had softening or fissuring lesions of the articular cartilage that needed any treatment.

**Table 1:** Comparison of Background Factors of Patients Between groups I and II^a^

Background Factors	Group I (*N*=44)	Group II (*N*=53)	P value
Mean age in years (range)	27 (14–57)	26 (15–50)	0.5347
Male/female ratio	30:14	31:22	0.3253
Mean interval between injury and operation (month)	14	16	0.3844
Meniscal injury (partial meniscectomy/repair)	12/2	10/5	0.4522
Mean height in cm (standard deviation)	167(8)	170(9)	0.0890
Mean mass in kg (standard deviation)	66(11)	67(11)	0.6568

^a^There were no significant differences between the 2 groups. Group I. 40N-40N at 30° fixation group; Group II, 30N-30N at 10°fixation group

Each patient underwent clinical examinations at 2 years or more after surgery. In Group I, 5 out of 49 patients were lost during the follow-up period. Subsequently, the authors could examine 44 patients (89.8 %) at the final follow up. There were 30 men and 14 women with an average age of 27 years at the time of surgery (Table 1). In Group II, 5 patients out of 58 patients were during the final follow up period. Subsequently, the authors could examine 53 patients (91.4 %) at the final follow up. There were 31 men and 22 women with an average age of 26 years at the time of surgery. The follow-up period ranged from 24 to 72 months with an average of 29 months.

### Surgical procedure

**Fig. 1:**
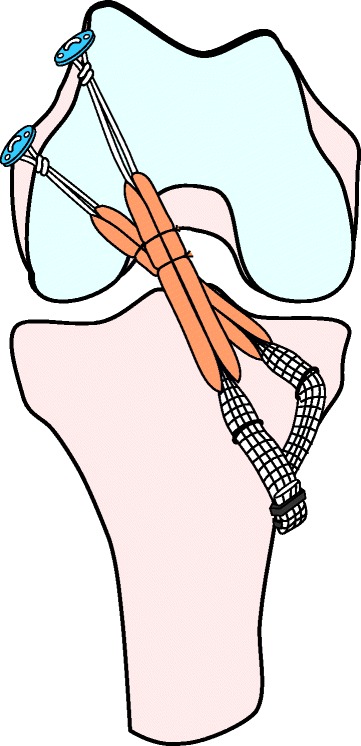
Schematic illustration of the anatomic double-bundle reconstruction

**Fig. 2:**
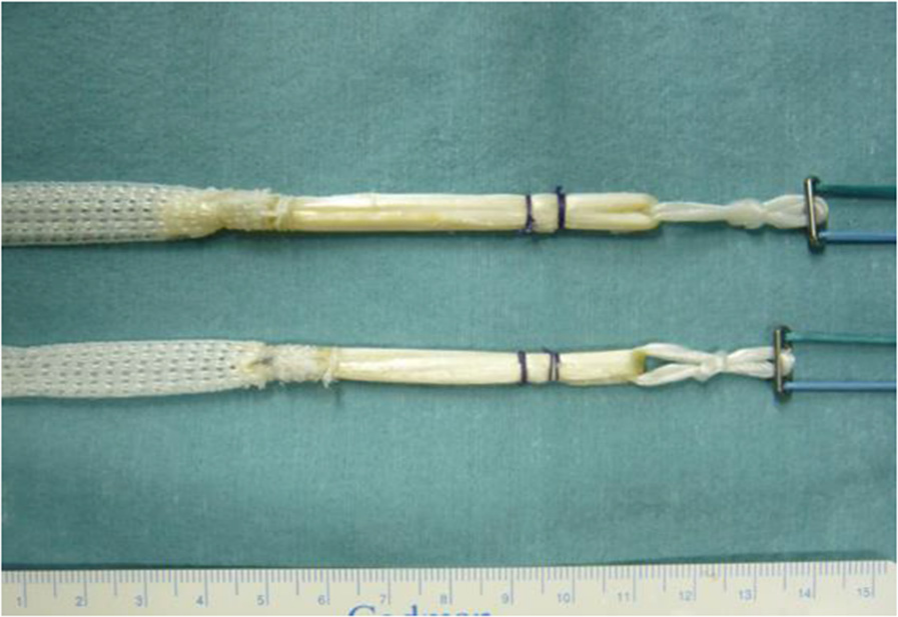
The hamstring tendon autografts were connected in series with polyester tape and EndoButton-CL-BTB (Smith & Nephew Endoscopy) for the double-bundle reconstruction

The details of the anatomic procedure were previously described in the literature (Fig. 1) [24, 25]. Using one-half of the semitendinosus and/or gracilis tendons, both free ends were firmly sutured, and used for the AM bundle graft. Commercially available polyester tape (Leeds-Keio artificial ligament, Neoligament, Leeds, England, United Kingdom) was then mechanically connected in series with the sutured end [21, 27]. Then, EndoButton-CL-BTB (Smith & Nephew Endoscopy, Andover, Massachusetts) was passed through the looped end portion of the tendon graft (Fig. 2). The remaining half of the semitendinosus tendon was also doubled, and the same type of fashioning was performed for the PL bundle graft (Fig. 2). The AM graft diameter ranged from 6 to 9 mm (mean, 7.1 ± 0.7 mm), and the PL graft diameter ranged from 5.4 to 7 mm (mean, 5.8 ± 0.4 mm).

**Fig. 3:**
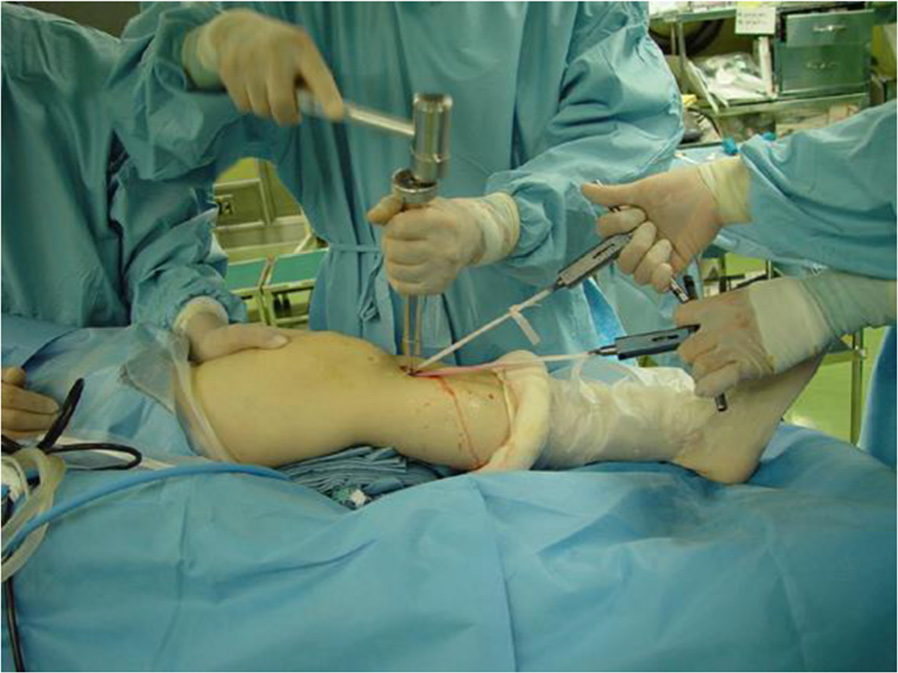
Graft tensioning methods. In Group I, the two tibial ends were simultaneously fixed at 30° of knee flexion with 2 spiked-staples, applying a total of 80 N load (a 40 N load to each graft) for 2 min using a custom-made spring-type tensiometer. In Group II, the two tibial ends were simultaneously fixed at 10° of knee flexion with same method, applying a total of 60 N load (a 30 N load to each graft)

First, a tibial tunnel for the PL and AM bundle was created using a Wire-navigator device (Smith & Nephew Endoscopy Japan, Tokyo, Japan). Then, the 2 tibial tunnels were made with a cannulated drill. To create 2 femoral tunnels for the AM and PL bundles in the lateral condyle, first a guidewire was drilled at the center of the femoral footprint of the AM bundle using an 5-mm offset guide (Transtibial Femoral ACL Drill Guide, Arthrex, Naples, Florida) inserted through the AM tibial tunnel. The surgeon drilled a guidewire at the center of the PL bundle attachment on the femur through the PL tibial tunnel. Finally, 2 sockets were created for the AM and PL bundles, respectively. The PL and AM grafts were introduced through the tibial tunnel to the femoral tunnel. Each femoral end was secured with an EndoButton (Smith & Nephew Endoscopy). In Group I, the 2 tibial ends were simultaneously fixed at 30° of knee flexion with 2 spiked-staples (Smith & Nephew Endoscopy), applying a total load of 80 N (a 40 N load to each graft) for 2 min using a custom-made spring-type tensiometer (Meira, Nagoya, Japan) (Fig. 3). In Group II, the 2 tibial ends were simultaneously fixed at 10° of knee flexion with same method, applying a total load of 60 N (a 30 N load to each graft).

### Postoperative management

The rehabilitation protocol was identical for both groups (Table 2). Postoperative management was performed according to the original rehabilitation protocol [20, 28]. A postoperative immobilizer was used for 2 weeks after the operation. Full weight bearing was then allowed with a hinged brace 2 weeks after surgery. Various kinds of athletic training were gradually allowed after 6 weeks, although no running was allowed until 9 months after surgery. Return to full sports activity was generally permitted at 12 months. The same rehabilitation protocol was used postoperatively for the patients who underwent the combined surgical treatments for torn menisci.

**Table 2:** Postoperative Rehabilitation Protocol^a^

Time Period (Postoperative)	Activities
Immediately	Walking (1/2 weightbearing) with an immobilization brace
	ROM exercise (0°-30°)
	Simultaneous quadriceps and hamstring (at 30° of knee flexion)
	Leg raising
	Reversed leg raising
	Hip abduction
2 weeks	Walking (full weight-bearing) with a functional brace
	ROM exercise (0°-120°)
	Separate isometric quadriceps (at 70° of knee flexion)
	Squatting at 90° of knee flexion
	Leg curl
4 weeks	Walking
	Half squat exercise (range, 70°-90°)
8 weeks	ROM exercise (0°-140°)
	Half squat exercise (range, 30°-90°)
	Fast walking
	Bicycling
	Calf raise
	Stairs exercise (walking)
12 weeks	ROM exercise (full range)
16 weeks	Isokinetic exercise (range, 30°-90°)
	Squat exercise (full range)
	Swimming (flutter kick)
20 weeks	Jogging
	Isokinetic contraction (full range)
9 months	Running, hopping, jumping rope
	Backward running, Carioca (lateral crossover)
12 months	Competitive sports

^a^ROM, range of motion

### Clinical evaluations

The side-to-side anterior laxity was measured with a KT-2000 arthrometer (MEDmetric, San Diego, California) at 30° of knee flexion under an anterior drawer force of 133 N. An experienced physical therapist who was not a coauthor of this study and blinded to the procedure collected the KT-2000 results postoperatively. One experienced orthopaedic surgeon (E.K.) who was blinded to the procedure performed the pivot-shift test, the results of which were subjectively evaluated by the examiner. In evaluation of the pivot-shift test, the result of test was consider 2+ when the examiner felt a sudden rotational slip movement between the tibia and femur, a so-called jog. A 2+ pivot-shift showed an obvious failure of the ACL function. The indication for + was when the examiner felt some difference in the rotational movement during the test between the injured and uninjured knees, but did not obviously feel the sudden rotational slip movement. This condition suggested some insufficiency of the ACL function but did not show a complete failure of the ACL. For overall evaluation, the Lysholm knee score (maximum score, 100 points) and the International Knee Documentation Committee (IKDC) form were used. Peak isokinetic torque of the quadriceps and the hamstrings were measured at 60° per second of angular velocity using Cybex II (Lumex, Ronkonkoma, Now York) in both knees before and after surgery. Muscle torque measured postoperatively in the uninvolved knee was represented as a ratio (percentage) to the uninvolved value.

### Statistical analysis

Statistical analysis was performed using StatView 5.0 (SAS Institute, Cary, North Carolina). Statistical comparison was made between the two groups using the unpaired Student *t* test and the chi-square test. Probability values less than 0.05 were considered indicative.

## Results

There wasn’t a significant difference in the background of the two groups (Table 1). There were no serious complications such as iatrogenic cartilage injuries, serious malposition of the tunnels, graft fixation failure during surgery, and no serious postoperative complications including fractures, deep vein thrombosis, and infections in any group.

After the final follow up, the postoperative side-to-side anterior laxity measured with the KT-2000 arthrometer averaged 1.1 mm and 0.9 mm in Groups I and II (Table 3), respectively. There was no significant difference between the two groups. Regarding the pivot-shift test, 11 and 12 patients were evaluated as ‘+’ and no patients were evaluated ‘2+’ in Groups I and II, respectively (Table 3). Evaluating postoperative range of motion revealed four patients (9 %) with loss of knee extension by 5–10° in Group I. No patient had loss of range of motion in Group II. In statistical comparison, the *p* value was 0.025, which showed a significant difference between the two groups. According to the Lysholm knee score, the IKDC evaluation, and the muscle strength, there were no significant differences between the two groups (Table 4).

**Table 3:** Comparisons of the Anterior Stability and the Pivot-shift test. (SD: standard deviation)

	Group I (N=44)	Group II (N=53)	P value
Mean side-to-side anterior laxity	1.1 mm (SD, 1.7)	0.9 mm (SD, 2.1)	0.6124
Pivot-shift test			0.7857
-	33 patients	41 patients	
+	11 patients	12 patients	
2+	0 patients	0 patients

**Table 4:** Comparisons in the Clinical Outcome between groups I and II (SD: Standard deviation, IKDC: International Knee Documentation Committee)

	Group I (N=44)	Group II (N=53)	P value
Loss of knee motion			0.025
Loss of extension (>5°)	4 patients (9%)	0 patients	
Loss of flexion (>15°)	0 patients	0 patients	
Mean Lysholm knee score (points)	98.1 (SD, 2.9)	97.5 (SD, 3.0)	0.3220
IKDC evaluation			0.4494
A (normal)	24 patients	34 patients	
B (nearly normal)	16 patients	17 patients	
C (nearly abnormal)	4 patients	2 patients	
D (abnormal)	0 patients	0 patients	
Mean isokinetic peak torque^a^			
Quadriceps muscle	87.4 % (SD, 11.8)	88.0 % (SD, 14.2)	0.8237
Hamstring muscle	95.8 % (SD, 15.3)	94.1 % (SD, 11.9)	0.5398

^a^Ratio of the treated knee to the uninjured knee, expressed as a percentage

## Discussion

The most important finding of this study was that, the 2 different initial graft tension protocols of anatomic double-bundle ACL reconstruction did not result in any significant differences concerning the postoperative anterior laxity, the muscle strength, the Lysholm knee score, and the IKDC evaluation. However, the authors found a significant difference in postoperative loss of knee extension between the two procedures. Namely, 9 % of the patients showed loss of knee extension by 5° - 10° in the former procedure, while there were no patients with loss of knee extension in the latter procedure. From the clinical viewpoint, the loss of knee extension is one of the pathological conditions that should be absolutely avoided after ACL reconstruction [29].

The fundamental biomechanical knowledge [5, 30, 31] would suggest that, when the 40-N initial tension was applied to each graft at 30° of knee flexion in the Group I, the graft tension might increase to 80 N or more at 10° of knee flexion immediately after surgery. This is significantly different from the graft tension at 10° of knee flexion in Group II, which was only 30 N in each graft. The authors speculate that the difference in the graft tensions at 10° of knee flexion may explain why there was some difference in postoperative loss of knee extension between the two procedures. Namely, the high tension in the 2 bundles, specifically in the PL graft, may increase the occurrence of flexion contracture. The authors speculation was that: The initial graft tension in hamstring tendon grafts is dramatically reduced by repetitive flexion-extension motion in the early phase after surgery, regardless of the type of fixation device [32, 33]. Therefore, the graft tension at each angle of knee flexion might decrease within the rehabilitation phase in the present study, and it made the difference at the final follow up examination less remarkable. Also it should be noted that the effects that rehabilitation could have on the grafts after 2 years is unclear. In the clinical field, however, it is known that the loss of knee extension should be avoided after ACL reconstruction [29]. Therefore, based on the present study, the authors do not recommend the initial graft tension protocol in which the 40-N initial tension was applied to each graft at 30° of knee flexion.

Biomechanical studies with cadaveric knees reported that an increase of ACL tension decreases the degree of anterior translation of the tibia underneath the femur [8, 31, 34, 35]. Therefore, it is considered that the knees in Group I were more overconstrained than the knees in Group II. Nevertheless, there was no significant difference in the anterior laxity between the two groups at the 2-year examination. The authors consider that this clinical result was also explained by the effect of the graft relaxation. Namely, the authors can say that the difference in the two graft tension protocols, a 40-N initial tension applied to each graft at 30° versus a 30-N initial tension applied to each graft at 10°, does not significantly affect the postoperative knee laxity in anatomic double bundle ACL reconstruction.

There have been a few biomechanical reports with cadaveric knees, in which a few initial graft tension protocols were compared [36–38]. Vercillo et al [38] conducted a double-bundle ACL reconstruction with both grafts fixed at 15° of knee flexion and again with the AM and PL grafts fixed at 45° and 15° of knee flexion. Each graft was fixed while a 67-N posterior tibial load and 22 N of initial graft tension were maintained. They concluded that knee flexion angles between 15° and 45° for graft fixation were found to be safe for the AM graft, while 15° of knee flexion was safe for the PL graft. Although these biomechanical studies provided important information on the effect of initial graft tension protocols, they did not provide any information to the final clinical outcome. Surprisingly, there had been no clinical studies in which two initial graft tension protocols were compared in patients who underwent the same anatomic double-bundle ACL reconstruction procedure. Therefore, the authors believe that the present study is of value in the clinical field.

There were some limitations to the present study. First, all patients for Group I were selected for an initial term of 2008 and 2009; and Group II from 2010–2011. However, no statistical differences were detected concerning the background factors between the two groups. Secondly, the authors compared only the 2 initial graft tension protocols. The authors cannot refer to other graft tension protocols. However, the 2 techniques compared are widely used around the world. Thirdly, at the time immediately after surgery, the authors did not measure the forces by which the authors could extend the knee to the full extension position. However, such measurements are not taken in common clinical examinations. Fourthly, the authors did not evaluate an accurate placement of the tibial and femoral tunnels for each patient using 3-dimentional computed tomography in this study. Although there were some limitations, the authors believe that the present study can provide useful information to the clinical field on ACL reconstruction.

## Conclusion

The 2 initial graft tension protocols did not result in any significant differences in the Lysholm knee score and IKDC grade. However, there was a significant difference that the 40-N tension applied to each graft at 30° of knee flexion more frequently induced loss of knee extension in a range between 5° and 10° in comparison to the 30-N tension applied to each graft at 10°. From the clinical viewpoint, the loss of knee extension is one of the pathological conditions that should be absolutely avoided after ACL reconstruction. Therefore, the 30-N tension applied to each graft at 10°is preferable to the other initial graft tension protocols.

## Abbreviations

ACL: anterior cruciate ligament
AM: anteromedial
PL: posterolateral
IKDC: International Knee Documentation Committee
